# Balancing Nutrition and Osmolality: Risk of Hyperosmolality During Individualized Fortification With Protein Fortifiers in an In Vitro Study

**DOI:** 10.7759/cureus.86602

**Published:** 2025-06-23

**Authors:** Laxman Basany, Abid Ali, Naga Priyanka G Gandrakota, Ajay B Kulkarni, Mahevish Tabassum, Harini Manjunath, Vinay Batthula

**Affiliations:** 1 Neonatology, Ankura Hospital for Women and Children, Hyderabad, IND; 2 Dietetics, Ankura Hospital for Women and Children, Hyderabad, IND; 3 Pharmacology, Independent Researcher, Bengaluru, IND

**Keywords:** breastmilk fortification, human milk fortifier, hyperosmolar, lipid fortifier, multicomponent fortifier, osmolality, preterm, protein fortifier, targeted fortification

## Abstract

Background: Individualized fortification of human milk with protein fortifiers (PFs) and fat fortifiers (FFs) helps optimize the nutritional requirements of preterm infants but increases osmolality. This study aims to evaluate the impact of PFs and FFs on the osmolality of human milk fortified with multi-component fortifiers (MCFs).

Methods: The osmolality of 25 mL of expressed breast milk (EBM) was measured with six MCFs (1 g each), preterm formula (PTF), PF, and FF separately. Additionally, the osmolality of 25 mL of sterile water was measured with 1 g of each MCF separately. PF was added in increasing amounts (0.5, 1, 1.5, and 2 g) to fortified human milk (FHM), and the maximum amount of PF that could be added without exceeding the osmolality of 450 mOsm/kg was determined.

Results: The osmolality of EBM was 288 mOsm/kg, which increased to 384 mOsm/kg with the addition of 1 g of PTF. Among FHM, the highest osmolality was observed with MCF4 (428 mOsm/kg), and the lowest with MCF6 (327 mOsm/kg). The addition of 0.5 g of PF to FHM with MCF1 and MCF4 increased osmolality beyond the safe threshold but was within safe limits with MCF2, MCF3, MCF5, and MCF6. When 1 g of PF was added to FHM with MCF2 and MCF3, osmolality exceeded 450 mOsm/kg. However, osmolality remained within safe limits with the addition of 1.5 g of PF to FHM with MCF5 and MCF6. The addition of 1 g of FF did not alter the osmolality of EBM.

Conclusion: The addition of MCFs increases the osmolality of EBM, although within safe limits. However, as the addition of PF to FHM further raises the osmolality, it is essential to select the type of MCF and adjust the amount of PF to maintain osmolality within safe threshold limits.

## Introduction

Breast milk is the ideal choice for neonates due to its nutritional, bioactive, and immunological components, which provide both short-term and long-term benefits [1]. Very-low-birth-weight (VLBW) infants have elevated nutritional requirements, making them vulnerable to extrauterine growth restriction, which is associated with an increased risk of complications such as retinopathy of prematurity (ROP), bronchopulmonary dysplasia (BPD), impaired renal function, and neurodevelopmental delays [2]. Although human milk (HM) is ideal, it lacks adequate protein, calcium, phosphorus, and micronutrients, necessitating fortification with multicomponent fortifiers (MCFs) to optimize the growth of VLBW infants [3,4]. Instead of MCFs, preterm formula (PTF) is often used to fortify HM in low-resource settings [5]. However, fortifiers can increase the osmolality of HM, leading to feeding intolerance, delayed gastric emptying, and an increased risk of necrotizing enterocolitis (NEC). Hence, the European Society for Paediatric Gastroenterology, Hepatology and Nutrition (ESPGHAN) and the American Academy of Pediatrics (AAP) recommend that the osmolality of enteral feeds should not exceed 450 mOsm/kg (400 mOsm/L) to minimize potential risks [6,7].

Standard fortification involves adding 1 g of MCF to 25 mL of HM, based on early lactation protein levels of 1.4-1.5 g/dL. However, as protein content declines to ≤1 g/dL by 4-6 weeks postpartum, this method often leads to inadequate protein intake and growth faltering seen in one-third of VLBW infants [3,8-11]. Henriksen et al. reported that 58% of VLBW infants experienced poor growth with standard fortification [12]. Standard fortification often falls short of meeting the protein needs of VLBW infants, providing only 2.8-2.9 g/kg/day instead of the recommended 3.5-4 g/kg/day [3,8].

Hence, the European Milk Bank Association (EMBA) advocates for "individualized fortification" to ensure optimal nutrient intake for preterm infants. The two approaches to individualized fortification are adjustable fortification and targeted fortification [3,8,12,13]. Adjustable fortification is a strategy where extra protein is added to fortified HM (FHM) based on the blood urea nitrogen (BUN) value. Targeted fortification involves analyzing HM and supplementing it with precise amounts of protein, lipids, and carbohydrates to meet specific nutritional goals [3,8,13]. Adjustable fortification has been shown to significantly improve growth parameters compared to standard fortification. Studies have shown a linear correlation between the osmolality of fortified milk and the quantity of added macronutrients [14,15]. Individualized or adjustable fortification of HM is increasingly used in neonatal intensive care units (NICUs) to meet the dynamic nutritional requirements of preterm infants. However, the addition of protein supplements to already FHM can significantly raise osmolality, potentially leading to feeding intolerance or intestinal injury. Despite the growing adoption of this practice, data on osmolality changes with adjustable fortification using different fortifiers available in India are lacking.

In this in vitro study, we have evaluated six commercially available HM fortifiers. Despite the widespread use of various HM fortifiers in India, there is a lack of data on how different fortifier types, such as HM-based, bovine-based, and plant-based, interact with incremental protein supplementation in terms of osmolality changes. The protein fortifier (PF) was added incrementally (0.5, 1.0, 1.5, and 2.0 g) to HM fortified with each product, and osmolality was measured at each stage.

A new amino acid-based PF, NeoPF (Analeptik Biologicals LLP, Bengaluru, India; FSSAI no. 11223334000608), and a fat fortifier (FF), NEO-LIPIDZ (Analeptik Biologicals LLP; FSSAI no. 11223334000608), were recently introduced in India. Each gram of NeoPF contains 0.75 g of protein, while each gram of NEO-LIPIDZ provides 9 kcal. This is the first in vitro study from India aimed at evaluating the effects on the osmolality of FHM supplemented with different MCFs from bovine, human, and plant-based sources, along with FFs and PFs available in India.

## Materials and methods

This in vitro study was approved by the hospital ethics committee. Osmolality was measured using the freezing point depression method with an osmometer (Osmomat 3000, Gonotec GmbH, Germany), which has a precision of 2 mOsm/kg. The osmometer was calibrated, and all tests were conducted by a technician who was blinded to the MCFs, PTF, PF, and FF used in the study. All measurements were performed in duplicate, with the two readings averaged. If the osmolality difference exceeded 2 mOsm/kg, a third measurement was taken, and the average of the two closest readings was taken.

The expressed breast milk (EBM) used in the study was collected, with informed consent, from a mother who had delivered a preterm infant at 32 weeks of gestation, two weeks prior to the study. The EBM samples, stored at 4°C, were thawed to room temperature using tepid water, and osmolality was measured 10 minutes after thawing. The study included six MCFs: MCF1 NeoLact MMF Plus (Neolacta Lifesciences Private Limited, Jigani, India), a human milk-derived fortifier; MCF2 Lactodex-HMF (Raptakos Brett & Co. Ltd, Mumbai, India), MCF3 PreNAN (Nestlé, Vevey, Switzerland), MCF4 NiQu HMoF (NeoWinn Biotech Private Limited, Bengaluru, India), and MCF5 AB-HMF (Analeptik Biologicals), which are bovine milk-based fortifiers; and MCF6 HMF Advance (Analeptik Biologicals), a plant-based fortifier whose chemical composition is provided in Table 1.

**Table 1:** Comparison of Multicomponent Fortifiers. HMF: human milk fortifier; MCT: medium-chain triglycerides; MCF: multicomponent fortifier; MMF: mother’s milk fortifier *Not mentioned by the manufacturer **Osmolality of 1 g of fortifier with 25 mL of expressed breast milk (EBM) measured at 10 min

Nutrients per 1 g	MCF1 NeoLact MMF Plus	MCF2 Lactodex-HMF	MCF3 PreNAN	MCF4 NiQu HMoF	MCF5 AB-HMF	MCF6 HMF Advance
Energy (kcal)	3.89	3.37	4	3.4	4	4.50
Protein (g)	0.27	0.27	0.3	0.3	0.3	0.35
Carbohydrate (g)	0.62	0.49	0.4	0.5	0.41	0.24
Fat (g)	0.036	0.04	0.2	0.04	0.1	0.18
Iron (mg)	0.085	0.03	0.36	0.4	0.32	*
Sodium (mg)	2.2	1.9	7.34	4.5	6.2	*
Calcium (mg)	5.98	15.8	15.9	15	15	20
Phosphorus (mg)	1.36	7.9	8.76	8	8	10
Osmolality** (mOsm/kg)	418	365	379	428	334	327
Source	Human milk	Bovine	Bovine	Bovine	Bovine	Plant

One gram of each of the six MCFs was added separately to 25 mL of EBM, and osmolality was measured 10 minutes after reconstitution. Additionally, osmolality was assessed after adding 0.5 and 1 g of PF (NeoPF) to 25 mL of FHM. Additionally, 1.5 and 2 g of PF were added separately to FHM containing MCF5 and MCF6, and osmolality was measured. The osmolality of 25 mL of EBM was measured after adding 1 and 2 g of PTF (Aptamil Preterm, Danone Nutricia, Utrecht, the Netherlands). The osmolality of 25 mL of sterile water was measured with 1 g of each MCF separately.

The osmolality of 25 mL of EBM was measured after adding 1 g of FF (NEO-LIPIDZ), which contains 10 mg of docosahexaenoic acid, 15 mg of alpha-linolenic acid, 10 mg of linoleic acid, and 0.75 g of medium-chain triglycerides, providing 9 kcal/g. The maximum amount of PF that could be safely added to FHM without exceeding an osmolality of 450 mOsm/kg was determined separately for each MCF.

Statistical analysis

Friedman’s test was used to compare the osmolality of the mixture of FHM, with six different MCFs and PFs (0.5 or 1 g). All pairwise comparisons were performed after the Dunn-Bonferroni correction. The Mann-Whitney U test was used to compare the osmolality of FHM with MCF5 vs. MCF6 with PF (1.5 or 2 g). p-value < 0.05 was considered statistically significant. DATAtab Statistic Calculator (DATAtab e.U. Graz, Austria) was used for statistical analysis.

## Results

**Figure 1:**
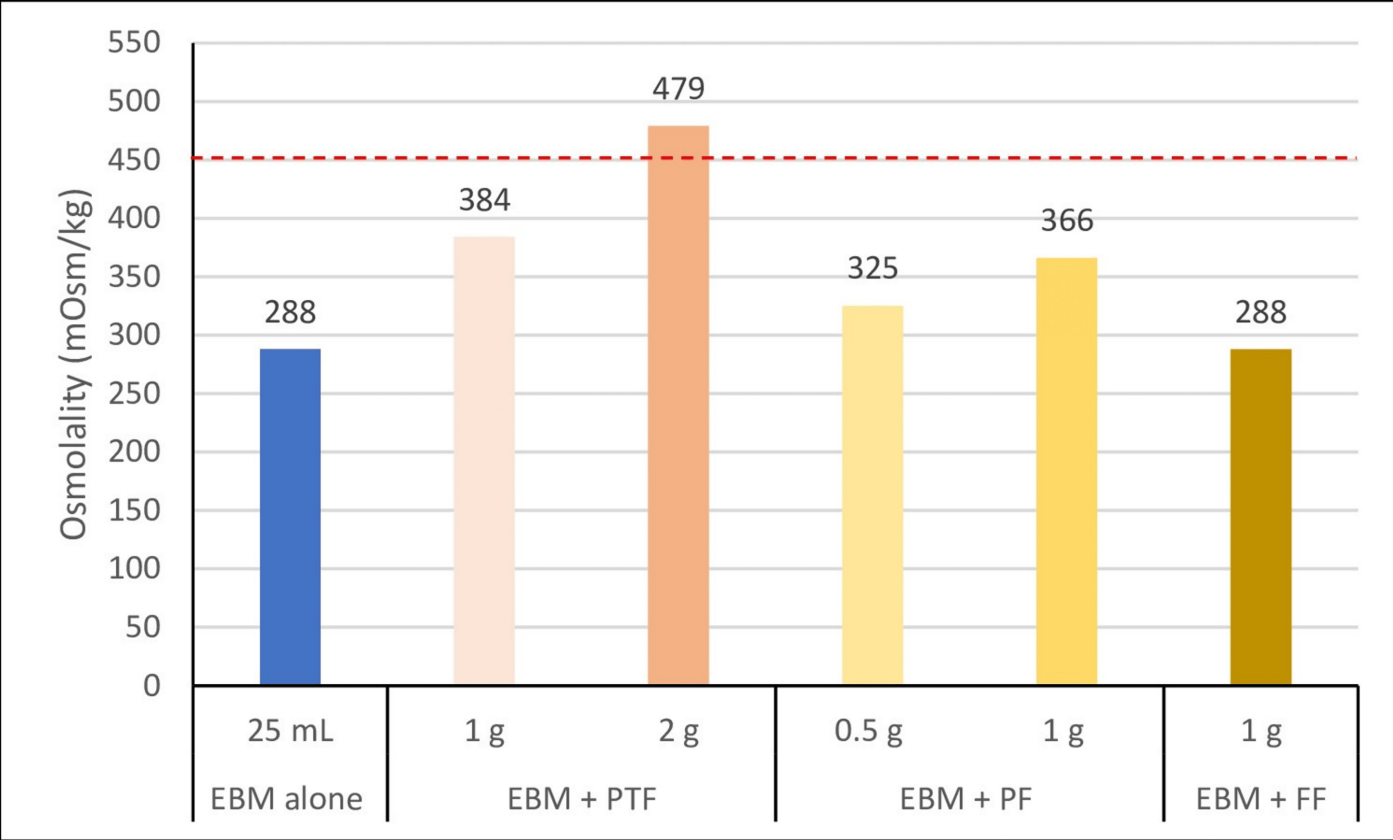
Osmolality of 25 mL EBM Fortified With PTF and Mono-Component Fortifier (PF and FF) Measured Individually. EBM: expressed breast milk; FF: fat fortifier; PF: protein fortifier; PTF: preterm formula

The mean osmolality of EBM was 288 mOsm/kg, ranging from 287 to 289 mOsm/kg. The addition of 1 and 2 g of PTF to EBM raised the osmolality to 384 and 479 mOsm/kg, respectively. The osmolality of EBM with 1 g of PF was 366 mOsm/kg. Figure 1 depicts the osmolality of 25 mL EBM with PTF (1 and 2 g), PF (0.5 and 1 g), and FF (1 g), measured separately.

**Figure 2:**
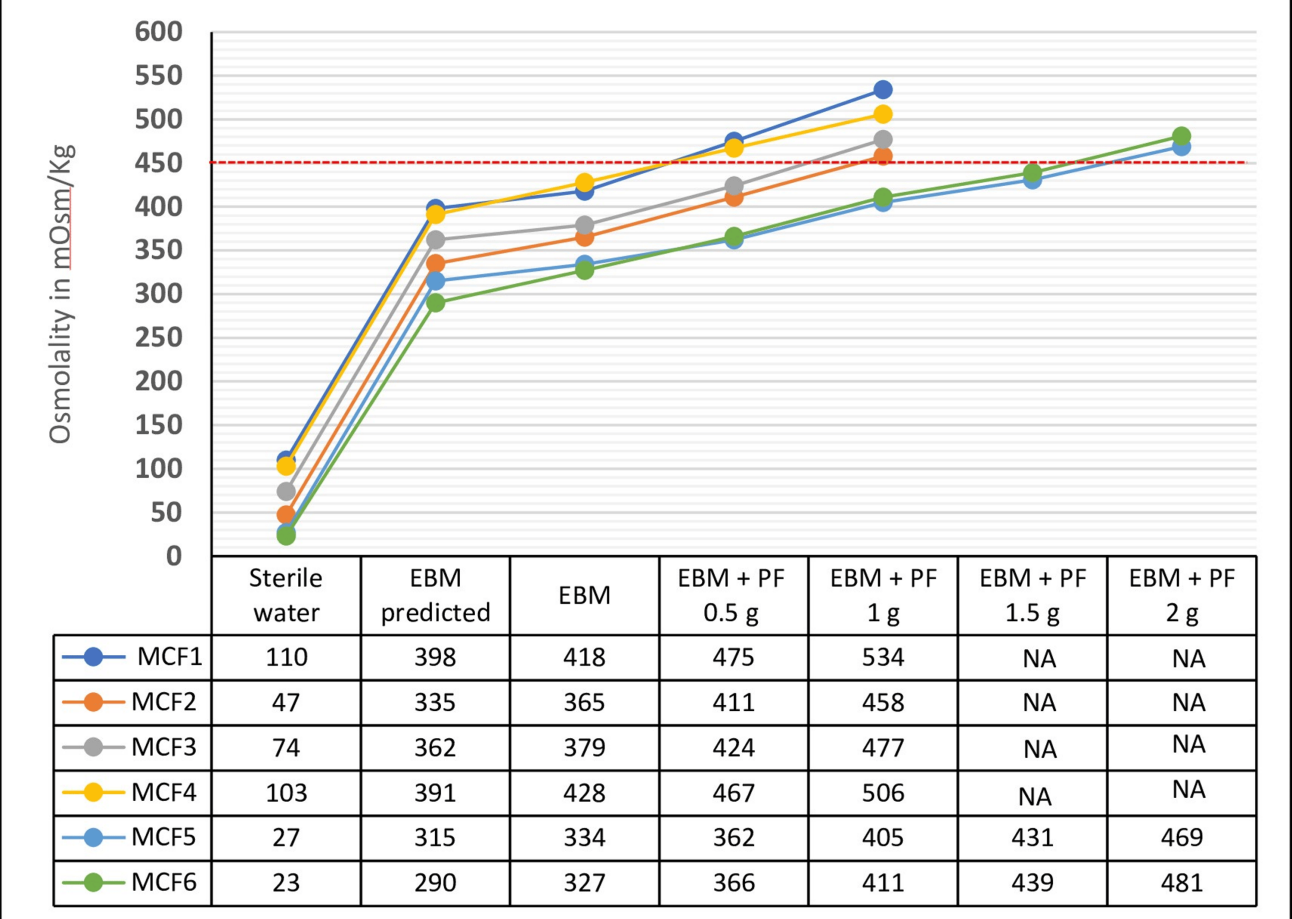
Measured Osmolality of MCF in Sterile Water, EBM with MCF and PF. EBM: expressed breast milk; HMF: human milk fortifier; MCF: multicomponent fortifier; PF: protein fortifier; MCF1: NeoLact MMF Plus; MCF2: Lactodex-HMF; MCF3: PreNan; MCF4: NiQu HMoF; MCF5: AB-HMF; MCF6: HMF Advance. Predicted osmolality is the osmolality measured of sterile water + osmolality of EBM. The osmolality of EBM alone observed was 288 mOsm/kg. PF used is NeoPF.

Figure 2 shows the osmolality of sterile water with MCFs and EBM with MCFs and PF, respectively. Figure 2 also shows the predicted and measured osmolality of EBM with the addition of MCFs. Predicted osmolality was calculated by adding the osmolality of the sterile water used for dilution to the baseline osmolality of EBM, which is 288 mOsm/kg.

The addition of MCFs increased the osmolality of HM, ranging from 327 to 428 mOsm/kg. Among the six MCFs tested, MCF6 caused the least increase in osmolality (327 mOsm/kg), followed by MCF5 (334 mOsm/kg), MCF2 (365 mOsm/kg), MCF3 (379 mOsm/kg), MCF1 (418 mOsm/kg), and MCF4 (428 mOsm/kg).

The addition of 0.5 g of PF to EBM fortified with MCF1, a human milk-derived fortifier, and MCF4, a bovine milk-based fortifier, resulted in osmolality levels of 475 and 467 mOsm/kg, respectively, which exceeded the safety limits.

The addition of 0.5 g of PF to EBM fortified with MCF2 and MCF3 raised the osmolality to 411 and 424 mOsm/kg, respectively, and with the addition of 1 g of PF, the osmolality increased to 458 and 477 mOsm/kg, exceeding the safety limits. There was no significant difference in the osmolality between FHM with six individual MCFs and FHM with six different MCFs plus PF (0.5 or 1 g, p = 0.059).

The addition of 0.5, 1, and 1.5 g of PF to FHM with MCF5 increased osmolality levels to 362, 405, and 431 mOsm/kg, respectively, which are within the safe limits. Similarly, the addition of 0.5, 1, and 1.5 g of PF to FHM with MCF6 increased osmolality to 366, 411, and 439 mOsm/kg, respectively, which were within the safe limits. However, the addition of 2 g of PF to HM fortified with MCF5 and MCF6 increased osmolality to 469 and 481 mOsm/kg, respectively, which exceeded the safe limits. The osmolality of the EBM with 1 g of FF remained the same at 288 mOsm/kg.

## Discussion

HM is considered the gold standard for feeding all neonates, including preterm infants. However, it does not provide sufficient nutrition to meet the needs of VLBW infants, necessitating fortification with MCFs to ensure adequate postnatal growth [3,8,10,11].

Standard fortification using MCF fails to address the variability in the macronutrient content of HM, often resulting in inadequate provision of macronutrients [3,10-12]. Individualized fortification, which includes adjustable and targeted fortification, meets the nutritional needs of preterm infants more effectively than standard fortification [3,8]. Adjustable fortification relies on monitoring BUN levels to adjust protein intake, whereas targeted fortification involves analyzing the nutrient content of HM and supplementing it with protein and/or energy to achieve the recommended nutrient levels [10,11]. Fortification of HM improves postnatal growth rates, but it increases the osmolality of the milk, which could result in potential adverse effects [14,15].

Srinivasan et al. documented a significant increase in osmolality, reaching up to 951 mOsm/kg, when medications and MCFs were used [16]. Similarly, Agarwal et al. noted a marked rise in the osmolality of HM with the addition of MCF and PTF [17].

Herranz Barbero et al. demonstrated that the osmolality of HM increased to 480 mOsm/kg with MCF, protein, and carbohydrate additives [15]. Similarly, Gupta et al. reported a rise in osmolality with MCF [18]. In addition, Pineda et al. found that the osmolality of FHM surpassed 450 mOsm/kg when a liquid protein supplement was used [19]. Kreins et al. reported a rise in osmolality when two PFs were added to FHM [20]. Choi et al. demonstrated a linear correlation between the osmolality of MCF and its macronutrient composition [14]. Recently, Basany et al. reported a notable rise in osmolality of pasteurized donor HM (PDHM) with the use of vitamins, minerals, additives, and MCFs, underscoring the need for appropriate dilution to prevent hyperosmolality [21].

The addition of MCFs to EBM raised the osmolality, which ranged from 327 to 428 mOsm/kg. Among the six MCFs evaluated, MCF6 resulted in the smallest rise in osmolality (327 mOsm/kg), followed by MCF5 (334 mOsm/kg). The highest osmolality was observed with MCF4 (428 mOsm/kg) followed by HM-based fortifier MCF1 (418 mOsm/kg).

The relatively low osmolality associated with MCF6 is likely due to its lower carbohydrate content of 24 g/100 g (Table 1). In contrast, MCF1 and MCF4 contain HM oligosaccharides (HMOs), which provide nutritional benefits but contribute to higher osmolality.

EBM fortified with MCF1 or MCF4 exceeds safe osmolality limits when additional protein is added-rising above 450 mOsm/kg with 0.5 or 1 g supplements. This highlights the risk of hyperosmolality with individualized fortification using these fortifiers. Adding 0.5 g of protein supplement to FHM with MCF2 and MCF3 keeps osmolality within safe limits (411-424 mOsm/kg), but 1 g exceeds the 450 mOsm/kg threshold. In contrast, FHM fortified with MCF5 and MCF6 maintains safe osmolality (≤450 mOsm/kg) even with protein supplements up to 1.5 g.

The discrepancy observed between the predicted and observed osmolality could be due to the effect of amylase on the carbohydrates present in the fortifiers and milk, as depicted in Figure 2. The differences in osmolality among the fortifiers can be explained by variations in the types of carbohydrates used, the concentration of oligosaccharides, and dextrins [21].

PTF is often used for the fortification of HM in low-resource settings [22]. FHM has a higher osmolality than PTF, which has an osmolality of around 300 mOsm/kg. Chinnappan et al. in their noninferiority trial, concluded that the growth of preterm infants receiving fortification with PTF was comparable to MCFs, with the benefit of less feed intolerance and lower cost [5]. Therefore, we evaluated the osmolality of EBM fortified with PTF and found that adding 1 g of PTF increased osmolality to 384 mOsm/kg, which is within safe limits. However, the addition of 2 g of PTF increased the osmolality to 479 mOsm/kg, which exceeded the recommended threshold. Pillai et al. used concentrated PTF as a fortifier in preterm infants and found that the liquid fortifier was better tolerated compared to powder-based alternatives [23].

Although FHM can be stored at 4°C for up to 72 hours, using it immediately after preparation is preferred to minimize the risk of microbial contamination. Osmolality increases over time due to carbohydrate breakdown; hence, fortifiers should be added just prior to feeding to minimize this effect. Hyperosmolar feeds and enteral medications can damage the intestinal mucosa, promote harmful bacteria, and lead to complications like osmotic diarrhea, intestinal ischemia, and delayed gastric emptying [24].

In a study conducted by Lucas et al. involving 275 preterm infants, the incidence of NEC was found to be higher in the group receiving fortified milk compared to the control group fed unfortified HM [25]. However, the current evidence from the literature does not strongly support a direct link between feed osmolality and gastrointestinal injury [26].

In our study, the addition of 1 g of FF did not alter the osmolality of the milk. In contrast, Rochow et al. found that an HM fortifier (MCF) containing lipids increased the osmolality of HM from 295 to 405 mOsm/kg, which further rose to 436 mOsm/kg following targeted fortification [27]. On the other hand, Choi et al. reported a reduction in osmolality when a fat supplement was added to HM [14]. These findings suggest that the energy content of HM can be safely enhanced by adding lipids without significantly affecting osmolality. The study demonstrated that adding PF to FHM with various MCFs significantly increased the osmolality often exceeding the safe thresholds recommended by ESPGHAN and AAP. However, judicious fortification helps preterm infants gain the benefits of HM while minimizing hyperosmolality risks.

With the exception of MCF4, none of the other five fortifiers-including the PFs and FFs-had osmolality information listed on their packaging. Although the product information for MCF4 listed the osmolality as <400 mOsm/kg, our measured value was 428 mOsm/kg, exceeding the declared limit. It is essential that all manufacturers clearly state the osmolality on the product label and provide guidance on appropriate dilution, particularly when additional protein is added. Notably, two fortifiers were found to be unsuitable for adjustable fortification due to a marked rise in osmolality. In contrast, the plant-based fortifier demonstrated the lowest osmolality across all levels of protein supplementation.

Our findings underscore the critical need for caution during adjustable fortification, as osmolality varied significantly between fortifier types. This study fills an important knowledge gap and provides practical guidance for safer, evidence-based nutritional strategies in the NICU.

Limitations of the study

We did not assess the impact of adding vitamins and other supplements on the osmolality of FHM, nor did we evaluate the clinical effects of hyperosmolar feeds on preterm infants. In our unit, fortifiers are added immediately before feeding, and fortified EBM is not stored in order to minimize the risk of contamination and prevent a potential rise in osmolality during storage. In this in vitro study, we used milk from a mother who had delivered a preterm infant at 32 weeks gestation two weeks prior. As the composition of HM is subject to individual variation, the results reported here reflect the specific characteristics of the milk from this single mother. Since EBM was not analyzed, the macronutrient content and caloric value remain unknown. Osmolality was measured 10 minutes post-fortification, so changes in osmolality with storage have not been evaluated. Furthermore, liquid fortifiers and carbohydrate fortifiers were not included in the study due to their unavailability in India.

## Conclusions

Fortification of HM with MCFs increases the osmolality, but it remains within the safe limit of <450 mOsm/kg. In contrast, PFs are often hyperosmolar, increasing osmolality beyond the safe limits. Educating caregivers on the correct use of fortifiers and protein and fat supplements minimizes potential adverse effects. Special caution is warranted when using MCF1 and MCF4 fortifiers for adjustable fortification, as osmolality levels exceed safe thresholds. FFs did not raise the osmolality of FHM, indicating their safe use in enhancing nutritional content without increasing the risk of hyperosmolality. Future research should focus on developing MCF and PF with lower osmolality to enhance safety in neonates. Additionally, the clinical implications of elevated osmolality levels remain unclear, highlighting the need for further investigation.
